# Insight into the Vacuolar Compartmentalization Process and the Effect Glutathione Regulation to This Process in the Hyperaccumulator Plant *Solanum nigrum* L.

**DOI:** 10.1155/2022/4359645

**Published:** 2022-04-29

**Authors:** Zhishuai Li, Wenjie Guan, Lan Yang, Yan Yang, Hongyan Yu, Luyi Zou, Yue Teng

**Affiliations:** ^1^School of Environmental and Civil Engineering, Jiangsu Key Laboratory of Anaerobic Biotechnology, Jiangnan University, Wuxi 214122, China; ^2^Jiangsu Collaborative Innovation Center of Technology and Material of Water Treatment, Suzhou 215009, China

## Abstract

Vacuole compartmentalization plays an important role in the storage of heavy metals in hyperaccumulators. Is the vacuolar compartmentation a simple shielding process or a dynamic process that continuously consumes cell sap resources? How does glutathione affect the process of vacuolar compartmentalization? These unknown questions are very important to understand the mechanism of vacuole compartmentalization and can provide a guide for the design of hyperaccumulator plants by genetic engineering. Therefore, this study explored the enzyme activities, total cadmium, Cd^2+^, glutathione, oxidized glutathione, and reactive oxygen species contents in protoplasts and vacuoles of leaf cells in *Solanum nigrum* L. through subcellular separation. The results showed that vacuolar compartmentalization was a dynamic process that actively induced the related substances produced by cell sap to enter the vacuole for detoxification. When regulating the decreased glutathione content with buthionine sulfoximine, the total cadmium and combined cadmium in protoplasm decreased significantly, but the vacuole still maintained a high proportion of cadmium content and stable ROS content, which indicated that various external resources were preferentially used to maintain cadmium storage and homeostasis in vacuole rather than outside vacuole. These findings could guide the use of genetic engineering to design hyperaccumulator plants.

## 1. Introduction

Cadmium (Cd) is a highly toxic and nonessential heavy metal in plants. Even at low concentrations, Cd can cause damage to plants at the molecular, subcellular, cellular, organic, and even whole plant level [1–4]. Different plants have different sensitivity to heavy metal exposure. In general, plants have developed two main strategies to resist exposure to high concentrations of heavy metals. One is the *exclusion strategy*, and the other is the *tolerance strategy* [5]. In hyperaccumulator plants, many studies on the accumulation and distribution of Cd at the cellular or subcellular level have shown that vacuoles play a major role in the storage and transportation of Cd [6]. In order not to damage the normal physiology of cells, hyperaccumulator plants mainly transfer and store Cd into vacuoles of above-ground cells (accounting for about 90%) for isolation. However, while storing a large amount of cadmium in the vacuole, whether it will induce oxidative stress in the vacuole and the relationship between the presence of cadmium and chelating detoxification substances such as glutathione produced outside the vacuole remains unclear. Understanding the influencing mechanism of external resource on internal consumption of vacuole compartmentalization and whether there is dynamic regulation in vacuole will be important for future genetic improvement of hyperaccumulator plants.

Different forms of cadmium have different toxic effects on plants. It is also worth studying how detoxification substances participate in the process of vacuolar storage of cadmium and how to maintain a dynamic balance. In plant cells, Cd^2+^ induces reactive oxygen species (ROS) production and consumes GSH [7]. GSH is a low-molecular weight mercaptan compound that plays a central role in the detoxification of Cd, including the formation of Cd-GSH complex, the synthesis of phytochelatins, and the removal of ROS [7–9]. Cd-GSH and Cd-PCs chelates can be transported to the vacuole via transporters on the vacuole membrane [10]. However, when a large amount of Cd is isolated in the vacuole, how do the GSH and ROS outside the vacuole react inside and outside the vacuole and whether there is a dynamic regulation process that induces the generation of GSH and ROS in the vacuole? Specifically, it is not clear whether the vacuole traps the Cd merely in the vacuole or whether there is a dynamic equilibrium storage of Cd caused by the coaction of the cytoplasm and the vacuole. Understanding these mechanisms is important to improve plant tolerance and enhance the phytoremediation effect. Therefore, we selected *Solanum nigrum* L. with large biomass, fast growth rate, and high Cd accumulation ability as the research object and simulated the natural environment in soil culture [11, 12]. The contents of Cd^2+^, total Cd, combined cadmium, GSH, GSSG, ROS, GR, GPX, and GST in single protoplasts and vacuoles were quantitatively determined to explore these problems.

In order to investigate the homeostasis regulation of GSH on the accumulation and storage of different forms of Cd in vacuoles, we also selected buthionine sulfoximine (BSO) as a specific inhibitor of GSH biosynthesis and studied it through controlled experiments. For this purpose, we obtained protoplasts and vacuoles by subcellular separation technology and quantified the content of Cd, Cd^2+^, and ROS in individual protoplasts and vacuoles using fluorescent probes [13, 14]. The variation trend of Cd^2+^ and ROS and the distribution of combined cadmium at different Cd concentrations were calculated and analyzed, and the accumulation and storage of vacuolar compartmentalization under different forms of cadmium were evaluated. In order to study the Cd chelation and antioxidation process affected by GSH during the compartmentalization of vacuoles, we also measured the changes of GR, GPX, GST enzyme activities, and GSH and GSSG content in single protoplasts and vacuoles. Through these studies, we can have a deeper understanding of the vacuolar compartmentalization process and how glutathione is utilized and consumed during this process and provide guidance for future genetic improvement of hyperaccumulator plants.

## 2. Materials and Methods

### 2.1. Plant Materials and Growth Condition

The seeds of *Solanum nigrum* L. were obtained from Panlong City, Sichuan, China. The seeds were soaked in 70% alcohol for 30 min, then treated with 2% NaClO_2_ for 10 min, and finally washed three times with ultrapure water. The seeds are evenly placed on the tray and cultivated for 5 days at a constant temperature of 26°C. After two weeks, the seedlings were transplanted into plastic flower pots (4 plants per pot). The soil was collected from the experimental field of Jiangnan University in Wuxi City, Jiangsu Province. The physical and chemical properties of the soil are shown in Table 1. The potting soil Cd concentration was set to three gradients (0, 20, and 40 mg kg^−1^) and 6 treatments (each treatment was repeated three times), including Cd0, Cd0 + BSO, Cd20, Cd20 + BSO, Cd40 and Cd40 + BSO. Cd was added to the soil in the form of a CdCl_2_ solution. Buthionine sulfoximine (BSO, glutathione biosynthesis inhibitor) was purchased from Sigma. After transplanting in greenhouse for 3 weeks, leaves were sprayed with 1 mmol L^−1^ BSO solution daily. After continuous spraying BSO for 1 week, complete leaves of *Solanum nigrum* L. were selected for the experiment.

### 2.2. Preparation and Purification of Leaf Protoplasts and Vacuoles

The extraction method of protoplasts is partially modified on the basis of Shen et al. [15]. Leaf samples (0.5 g) were collected and washed with ultrapure water. After absorbing the moisture on the leaf surface with a clean paper towel, the stems were removed, and the leaves were sliced into 2-mm^2^ pieces with a sharp razor. The leaves were placed in a petri dish (9 cm in diameter). Add 15 mL of 1.4% (w/v) Cellulase Onozuka R-10 (Yakult Honsha, Tokyo, Japan), 0.3% (w/v) Macerozyme R-10, 0.69 mol L^−1^ mannitol, 20 mmol L^−1^ 2-(N-morpholino) ethanesulfonic acid (MES), 0.5 mmol L^−1^ CaCl_2_, and 1 mol L^−1^ KOH to the protoplast enzymatic hydrolysate with pH 5.7 in the petri dish. Leaves were incubated in medium in a petri dish for 2 h in the dark at 27°C with 60 rpm shaking. After incubation, the suspension was quickly filtered with 240-mesh nylon mesh, and centrifuged for 5 min at 120 g with up and down acceleration of 3. After the first centrifugation, the supernatant was discarded, and the enzyme-free protoplast hydrolysate was added and centrifuged twice to eliminate the enzyme. Finally, 2 mL protoplast suspension was left for subsequent experiments.

The extraction of vacuoles was also partially modified in the method of Shen et al. [15]. The above cleaned 1 mL protoplast suspension was mixed with 10 mL protoplast lysate (0.9 mol L^−1^ mannitol, 0.8 mmol L^−1^ ethylene glycol tetraacetic acid (EGTA), 0.4 mmol L^−1^ 3-[(3-cholamidopropyl) dimethylammonio]-1-propanesulfonate (CHAPs), 20 mmol L^−1^ MES, and 2 mol L^−1^ Tris to adjust the pH to 7.5). Five minutes later, the mixture was incubated in 37°C warm water for 10 min and centrifuged at 900 *g* for 5 min. After the first centrifugation, the supernatant was discarded, and vacuolar washing solution (0.9 mol L^−1^ mannitol, 20 mmol L^−1^ MES, and 2 mol L^−1^ Tris, pH 7.5) was added to remove EGTA and CHAP_S_ by centrifugation twice. Finally, 1 mL of vacuole suspension was left for subsequent experiments.

### 2.3. Fluorescence Microscopy

In this experiment, a fluorescence microscope was used to obtain fluorescence images of relevant indicators, in which the excitation wavelength was set at 488 nm, and the emission capture wavelength was set at 525 ± 50 nm. The protoplasts and vacuoles were incubated with different dyes or probes to obtain fluorescent images, and the Cd^2+^ and ROS were quantified.

### 2.4. Determination of GR, GPX, and GST Enzyme Activities in Single Cells

In this experiment, the enzyme activity was determined by the kit of Nanjing Jiancheng Biology Co., Ltd.

#### 2.4.1. Determination of GR Enzyme Activity

The determination of GR enzyme activity was based on Zhang et al. [16]. Finally, the average enzyme activity of GR in a single protoplasm was calculated by the number of protoplasts in the suspension and the total enzyme activity.

#### 2.4.2. Determination of GST Enzyme Activity

The determination of GST enzyme activity was based on Zhang et al. [17]. Finally, the average enzyme activity of GST in a single protoplasm was calculated by the number of protoplasts in the suspension and the total enzyme activity.

#### 2.4.3. Determination of GPX Enzyme Activity

The determination of GPX enzyme activity was based on Zhang et al. [17]. Finally, the average enzyme activity of GPX in a single protoplasm was calculated by the number of protoplasts in the suspension and the total enzyme activity.

### 2.5. Determination of T-GSH, GSSG, and GSH Contents in Single Cells

In this experiment, the contents of T-GSH, GSSG, and GSH were determined by the kit of Nanjing Jiancheng Biology Co., Ltd.

#### 2.5.1. T-GSH Content Determination

The determination of T-GSH content was based on Zhang et al. [16]. Finally, the T-GSH content in a single protoplast (or vacuole) was calculated according to the number of protoplasts (or vacuoles) in the suspension and the total T-GSH content.

#### 2.5.2. GSSG Content Determination

The determination of GSSG content was based on Zhang et al. [16]. Finally, the GSSG content in a single protoplast (or vacuole) was calculated according to the number of protoplasts (or vacuoles) in the suspension and the total GSSG content.

#### 2.5.3. GSH Content Determination

Glutathione (GSH) content = T − GSH content − 2 × GSSG content.

### 2.6. Determination of Total cd in Single Cells

Atomic absorption spectrophotometer (AAS, AAF-7000 F, SHIMADZU, Japan) was used for determination. The 6 mL of protoplast suspension (or vacuole suspension) and 5 mL of HNO_3_ were added to the digestion tank and digested with microwave digestion apparatus. After the digestion, the HNO_3_ was removed by acidulation. The volume was fixed to 10 ml, and the determination was made with AAS. The total Cd content within a single protoplast (or vacuole) was calculated by measuring the total Cd content and the number of protoplasts (or vacuoles) in a 6 ml protoplast suspension (or vacuole suspension).

### 2.7. Microscopic Imaging and Quantification of Cd in Single Cells

In microscopic imaging and quantification of Cd, we use Cd specific probe Leadmium™ Green AM dyes (Thermo Fisher Scientific, USA). The dye solution was prepared in DMSO and diluted with 1 : 10 0.01 mol L^−1^ NaCl. The protoplast suspension (or vacuole suspension) obtained above was mixed with the stock solution at 0.5 final dye concentration and incubated for 2 h under dark conditions. After incubation, the suspension was cleaned with cleaning solution for 3 times, and subsequent measurements were made [18].

### 2.8. Microscopic Imaging and Quantification of ROS in Single Cells

In the determination of ROS on a single protoplast (or vacuole), we used a dihydrorhodamine 123 (DHR-123, Sigma-Aldrich, USA) probe. Probe reserve solution was prepared by dissolving 2 mg probe in 1 mL DMSO. High purity water was used to dilute 500 *μ*mol L^−1^ of probe working solution. The protoplast suspension (or vacuole suspension) obtained above was incubated with the probe at the final concentration of 10 *μ*mol L^−1^ in the dark for 20 min, and subsequent measurements were performed [19].

### 2.9. Fluorescence Image Analysis and Statistical Analysis

Image analysis was performed using ImageJ software. For counting, the liquid was collected 3 times, and 6 images were acquired each time. The average number of cells was taken as a data point. The data points and error bars represent the mean (*n* = 3) and standard deviation, respectively. Statistical analyses were performed using Origin 2018 and IBM SPSS 26 (ANOVA).

## 3. Results

### 3.1. Effects of Different Treatments on Protoplast Yield

**Figure 1:**
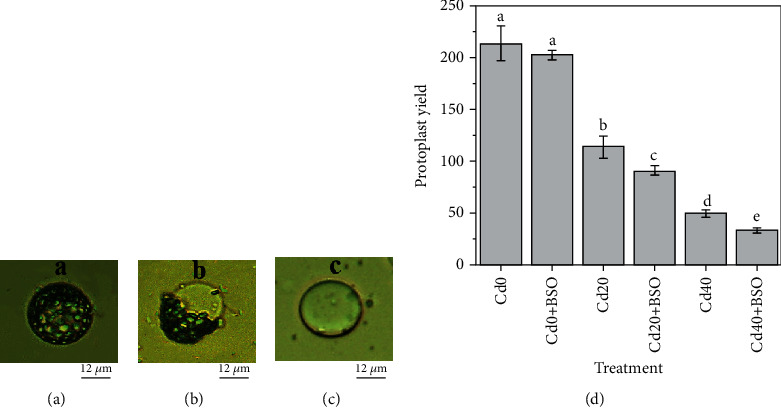
The protoplasts and vacuoles isolated from young leaves of *Solanum nigrum* L. and physiological characteristics of (a) protoplasts, (b) cleaved protoplasts, and (c) vacuoles. (d) Yield of protoplasts in leaves of *Solanum nigrum* L. on a single photomicrograph. Error bars represent standard error. Reported data are the mean ± standard error (SE) of at least three independent experiments, and different letters indicate significant differences between treatments at *P* < 0.05.

**Figure 2:**
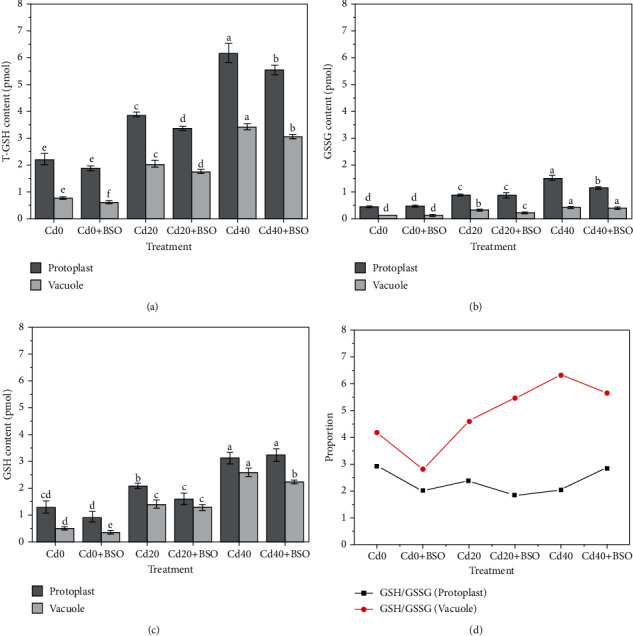
Effects of different Cd concentrations and BSO treatments on the contents and related proportions of T-GSH, GSSG, and GSH in protoplasts and vacuoles. (a) Changes of the average T-GSH content in a single protoplast and vacuole under different treatments. (b) Changes of the average GSSG content in a single protoplast and vacuole under different treatments. (c) Changes of the average GSH content in a single protoplast and vacuole under different treatments. (d) Average proportion curves of protoplasts and vacuoles under different treatments. Error bars represent standard error. Reported data are the mean ± standard error (SE) of at least three independent experiments, different letters indicate significant differences between treatments at *P* < 0.05.

**Figure 3:**
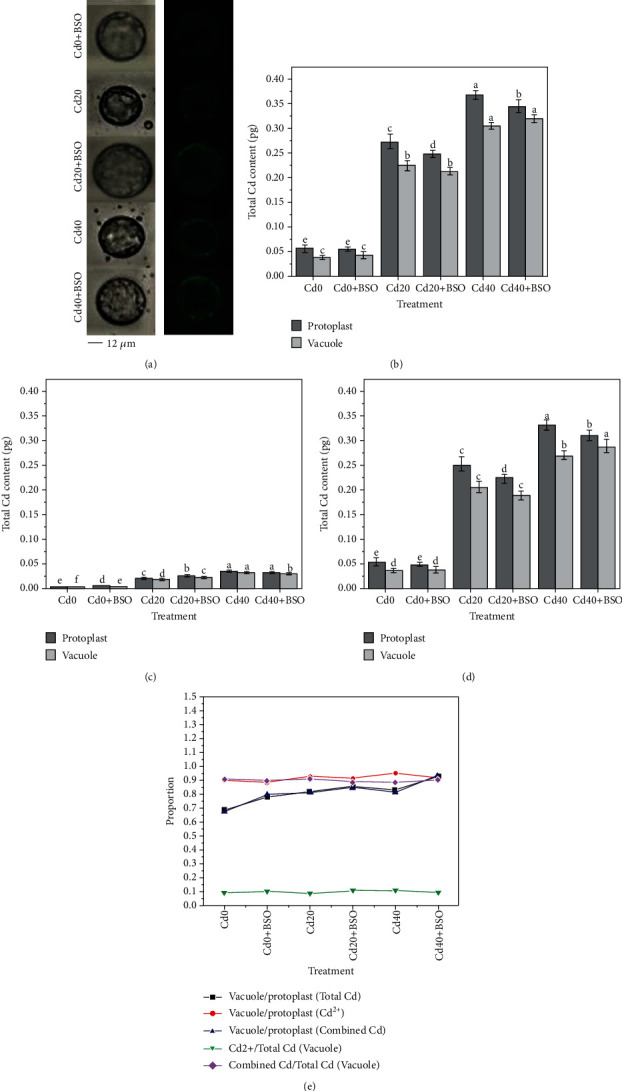
Effects of different Cd concentrations and BSO treatments on the contents and related proportions of total Cd, Cd^2+^, and combined Cd in protoplasts and vacuoles. (a) The photomicrographs of protoplasts in bright field and fluorescence field taken with probes under different treatments. (b) Changes of the average total Cd content in a single protoplast and vacuole under different treatments. (c) Changes of the average Cd^2+^ content in a single protoplast and vacuole under different treatments. (d) Changes of the average combined Cd content in a single protoplast and vacuole under different treatments. (e) Average proportion curves of protoplasts and vacuoles under different treatments. Error bars represent standard error. Reported data are the mean ± standard error (SE) of at least three independent experiments, and different letters indicate significant differences between treatments at *P* < 0.05.

**Figure 4:**
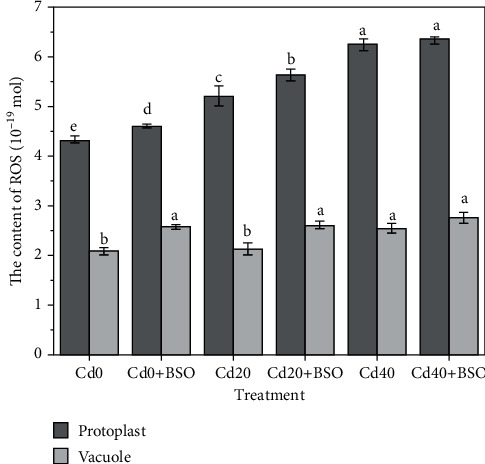
Effects of different Cd concentrations and BSO treatments on ROS content in protoplasts and vacuoles. Changes of the average ROS contents in a single protoplast and vacuole under different treatments. Error bars represent standard error. Reported data are the mean ± standard error (SE) of at least three independent experiments, and different letters indicate significant differences between treatments at *P* < 0.05.

Figures 1(a), 1(b), and 1(c) show the separated protoplasts, cleaved protoplasts, and vacuoles from the fresh leaves of *Solanum nigrum* L. The yield of protoplasts obtained under different treatment conditions is shown in Figure 1(d). With the increase of soil Cd concentration, the yield of protoplasts decreased, from 213 to 49 single images. The change of protoplast yield is because under high concentration of Cd, excessive Cd will be enriched in leaf cells of *Solanum nigrum* L., which will cause oxidative damage to cell membranes and internal cells, resulting in easy cell fragmentation. BSO treatment reduced protoplast yield, especially at Cd20 and Cd40 concentrations, compared with treatment without BSO at the same Cd concentration. The results showed that the reduction of GSH content caused by BSO would lead to the reduction of Cd chelation detoxification and antioxidant (Figures 2, Figure 3, and Figure 4), further resulting in oxidative damage of cells, and also proved the importance of GSH in the process of heavy metal accumulation in hyperaccumulator plants.

### 3.2. Effects of Different Treatments on GR, GPX, and GST Enzyme Activities in Protoplasts

**Figure 5:**
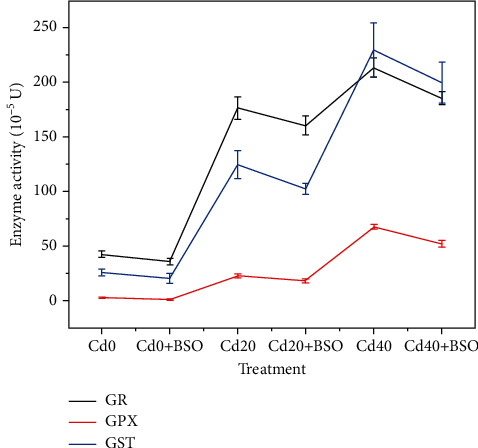
The enzyme activity curve of GR, GPX, and GST in *Solanum nigrum* L. leaf cell protoplasts under different Cd concentrations and BSO treatments. Error bars represent standard error. Reported data are the mean ± standard error (SE) of at least three independent experiments.

In order to further investigate the changes of glutathione pool and its effects on heavy metal chelation and antioxidant activity after exposure to Cd and BSO, we analyzed the enzymes related to its transformation metabolism, namely, GR, GPX, and GST. With the increase of Cd concentration, the activities of GR, GPX, and GST increased (Figure 5). The enzyme activity of GR and GST increased greater, which was more than 4 times that of the blank control at Cd40. This indicates that after a large amount of heavy metals are absorbed, a large amount of GSH and derivatives will be produced through the increase of enzyme activity in plant cells, thus maintaining the chelating storage and antioxidant detoxification of heavy metals. In contrast, the application of BSO altered the enzyme activities of GR, GPX, and GST. Changes in the activity of GSH-related enzymes will cause insufficient GSH pools in cells (Figure 2). When the concentration of Cd is high, it is not enough to reach the homeostasis mechanism of heavy metal chelation detoxification, and then the cells are prone to fragmentation and death (Figures 3 and Figure 4).

### 3.3. Effects of Different Treatments on the Contents of T-GSH, GSSG and GSH in Protoplasts and Vacuoles

In order to investigate the GSH-mediated Cd chelation, detoxification, and antioxidant processes at the single cell level of hyperaccumulator plants, we analyzed the changes in the contents of T-GSH, GSSG, and GSH in protoplasts and vacuoles. The changes of T-GSH, GSSG, and GSH contents are shown in Figures 2(a), 2(b), and 2(c). With the increase of Cd concentration in soil, the content of glutathione in protoplasts and vacuoles increased, and the proportion of GSH was the highest, indicating the importance of GSH in the cell to deal with heavy metal stress and detoxification. Compared with the control group, the contents of T-GSH, GSSG, and GSH in protoplasts and vacuoles of the BSO treatment group were decreased, and the tolerance to Cd was decreased.

In order to maintain the structure and function of the protein, the redox environment in the cell is essential. The GSH/GSSG ratio is an important indicator to reflect the redox state of plant cells.  Figure 2(d) and Table 2 shows the changes of various proportions. It can be found that the ratio of GSH/GSSG in vacuoles is higher than that in protoplast under different treatments, especially when the concentration of Cd in soil is Cd40, the vacuole can reach more than 3 times of the ratio in protoplast. This suggests that in order to prevent oxidative damage caused by large amounts of cadmium in vacuoles, hyperaccumulator plants expand the redox buffer by increasing the activity of various enzymes (such as GR) (Figure 5), thus sealing Cd to reduce damage. Similarly, this result also proves that in the process of Cd vacuole compartmentalization, the vacuole is not a single imprisoned Cd but a dynamic regulation process similar to an arena. Secondly, the proportions of vacuolar T-GSH in protoplasts were 34.82%, 52.38%, and 55.38%, and the proportions of vacuolar GSSG in protoplasts were 27.72%, 34.53%, and 26.86% under Cd (0, 20 and 40) treatments. The proportions of vacuolar GSH in protoplasts were 39.7%, 69.78%, and 76.01%. The increase in the content and proportion of GSH in vacuoles also indicates the importance of GSH for Cd chelation and detoxification during the dynamic regulation of vacuolar compartmentalization.

### 3.4. Microscopic Imaging and Quantification of Cd under Different Treatments

AAS and fluorescence calibration curves were used to determine the average contents of total Cd and Cd^2+^ in protoplasts and vacuoles. The combined cadmium content and various content ratios were calculated. It can be seen from Figures 3(b), 3(c), and 3(d) that with the increase of soil Cd concentration, the Cd content in protoplasts and vacuoles increased. In particular, Cd content in protoplasts and vacuoles of Cd20 and Cd40 increased compared with Cd0 treatments, indicating that the hyperaccumulator plant *Solanum nigrum* L. had a high Cd enrichment capacity. On the contrary, compared with the control, the Cd content in the protoplasts treated with BSO was reduced. BSO can decrease the content of GSH and combined cadmium in protoplasm of hyperaccumulator plants, while the ionic cadmium content is not affected, indicating that the reduction of GSH will lead to the decrease of the accumulation effect of cadmium in hyperaccumulator plants, and strengthening the overexpression of GSH will be conducive to the accumulation of cadmium in leaves.

As highly toxic Cd^2+^, plants are targeted to transport large amounts of Cd^2+^ into vacuoles to reduce cell damage, which proves that the vacuole is a storage area. The proportions of total and combined cadmium of vacuoles in protoplasts are 68.42%-92.73% and 67.23%-92.73% and gradually increase with the increase of Cd concentration. This also proves that Cd is mainly stored in vacuoles in the form of low-toxic combined cadmium, thus reducing the toxicity of Cd^2+^. Similarly, the proportion of Cd^2+^ in the vacuole to the total Cd ranged from 8.69% to 11.08%, and the proportion of combined cadmium to the total Cd ranged from 88.82% to 91.16%. These results suggest that glutathione content plays an important role in the accumulation of cadmium in hyperaccumulator plants and maintains a relatively stable state through resource regulation in vacuoles.

### 3.5. Microscopic Imaging and Quantification of ROS under Different Treatments

**Figure 6:**
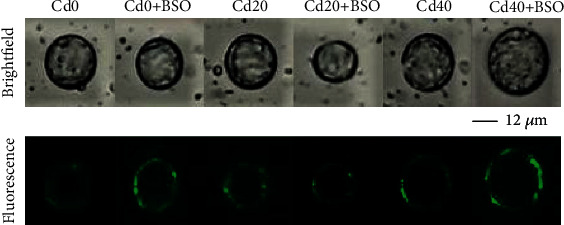
Effects of different Cd concentrations and BSO treatments on ROS content in protoplasts and vacuoles. The micrographs of protoplasts under different treatments in bright field and fluorescence field.

The contents of ROS in a single protoplast and vacuole were measured by the method of fluorescence image and calibration curve, as shown in Figures 6 and 4. With the increase of Cd concentration in soil, ROS content in protoplasm gradually increased, which was related to the amount of enriched Cd content. Compared with the control, ROS was also increased after BSO treatment, which was attributed to the inhibition of GSH production, resulting in a reduced chelation detoxification of Cd (Figure 2). Compared with the increase of ROS in protoplasts, the ROS in a single vacuole was maintained at about 2.5 × 10^−19^ mol. This result shows that under the increase of cadmium stress, cells well preferentially maintain the homeostasis in vacuoles by consuming glutathione.

## 4. Discussion

### 4.1. Vacuolar Compartmentalization Is a Dynamic Process That Consumes Resources outside the Vacuole rather than a Static Shielding Process

Hyperaccumulator plants accumulate large amounts of cadmium in above-ground parts when exposed to toxic cadmium concentrations. The majority of cadmium is sequestered in the vacuoles of leaf cells to maintain normal cellular physiology [20]. In the vacuole compartmentalization process, whether it is a simple storage of cadmium or the dynamic regulation process of continuously consuming internal and external resources of the vacuole is worthy of our intensive research. In this study, with the increase of cadmium stress, the total cadmium content in both single protoplast and vacuole increased, and 82.05%-82.83% of cadmium was stored in vacuole (Figure 3(e)). It is important to note that the change trend of glutathione and total cadmium content is similar (Figures 2(a), 2(b), and 2(c)), and the result indicates that the continuous consumption of glutathione in and out of vacuoles plays an important role in the process of chelation detoxification and vacuolar compartmentalization [21, 22]. By using the probe quantification method, we found that the most toxic ionic cadmium was isolated in the vacuole (89.92%-95.3%) (Figure 3(c)). When the ionic cadmium content in vacuole increased gradually, the content of glutathione outside the vacuole also increased (Figure 2), and the related enzyme activities also changed (Figure 5). Most of the increased glutathione was transported into the vacuole, and the proportion of GSH content in the vacuole reached 83.38% when Cd40 was treated (Table 2). The high GSH content in vacuoles is due to the maintenance of a high GSH/GSSG ratio to avoid the toxic effects of ionic cadmium (Figure 2(d)) [7, 23]. This indicates that ionic cadmium is not stored alone during vacuole compartmentalization but is actively transported into vacuoles for isolation [24, 25]. Compared with ionic cadmium, the ratio of less toxic combined cadmium to the total cadmium is very high, and the combined cadmium content in the vacuole accounts for 81.01%-83.89% of the protoplast (Figure 3). In vacuoles, the proportion of combined cadmium is as high as 90.85%-91.16% of total cadmium (Figure 3(e)), which also proves the importance of maintaining a high ratio of GSH/GSSG in vacuoles through resource regulation in reducing the toxicity of ionic cadmium by chelating detoxification [26]. With the increase of ionic cadmium content outside the vacuole, the induced ROS content also gradually increases (Figure 4), but this phenomenon does not appear in the vacuole. The results show that ROS content in a single vacuole has been maintained at an average of 2.5 × 10^−19^ mol, which is directly related to the high GSH/GSSG ratio in the vacuole. This also proves that vacuolar compartmentalization is a regulatory process that maintains homeostasis through constant consumption of resources [27, 28], which will provide guidance for considering the resources consumption in cell sap, when we design the genetic hyperaccumulator plants in the future.

### 4.2. Effect of Glutathione on the Process of Vacuolar Compartmentalization

According to the above research, we found that vacuolar compartmentalization was a dynamic process of homeostasis with consuming the resources in cell sap, and glutathione played an important role in this process. To further explore the influencing mechanism of glutathione on the vacuolar compartmentalization process, glutathione inhibitor BSO was used. The results showed that with the decrease of glutathione content, the total cadmium content in protoplasts and vacuoles also decreased (Figures 2 and 3), indicating that the regulation of glutathione content was related to the accumulation of cadmium in hyperaccumulator plant and high glutathione content could promote the accumulation of cadmium in hyperaccumulator plant cells [29]. Based on this relationship, we can enhance the expression of glutathione gene through genetic engineering, so as to obtain hyperaccumulator plants with higher accumulation capacity.

In the study of different forms of cadmium, we found that when regulating decreased glutathione content in protoplasm with BSO, the contents of ionic cadmium in protoplasts and vacuoles were not affected and kept unchanged. On the contrary, the content of combined cadmium showed the same decreasing trend as the total cadmium (Figure 3). These results indicated that the regulation of the decreased glutathione would not affect the accumulation of ionic cadmium but reduce the accumulation of combined cadmium, resulting in the decreased accumulation of the total cadmium in hyperaccumulator plant. Thus, if the glutathione gene is overexpressed, the accumulation of total cadmium in hyperaccumulator plants may enhance by increasing the accumulation of combined cadmium.

Based on previous studies and knowledge, hyperaccumulator plants sequestered heavy metal in leaf cell vacuoles to protect organelles and enzymes in cell sap, which indicated cells have a higher priority to protect the metabolism in cell sap (outside vacuole) [30–32]. However, this study showed that with the decrease of glutathione content in protoplasm, the content of ionic cadmium remained stable and was almost all stored in vacuole. It is worth noting that for highly toxic ionic cadmium, the interior of vacuole could still rely on a high GSH/GSSG ratio to maintain homeostasis and keep the content of ROS relatively stable through glutathione transport and consumption (Figure 2). Moreover, the regulation of decreased glutathione led to the increased content of ROS in the cell sap and weakened the protection of the metabolism in cell sap. This result is contrary to previous studies, and in the case of accumulation of a large amount of cadmium, cells do not have a priority to protect the environment outside the vacuole but have a priority to maintain the homeostasis in the vacuole through consumption of glutathione.

## 5. Conclusions

In summary, the results of this study clearly indicated that vacuolar compartmentalization was a process of continuous consumption of external resources to achieve dynamic regulation within the vacuole. With the increase of cadmium stress, a large amount of glutathione was produced in protoplasm, and the total cadmium content was increased in the form of a large increase in combined cadmium content. The high GSH/GSSG ratio is maintained in vacuole through the transport of GSH outside the vacuole, so as to cope with the high content of ionic cadmium and maintain the balance of ROS content. When regulating the decreased glutathione content with BSO, the total cadmium content decreased, which was due to the contribution of combined cadmium. The results indicated the cadmium accumulation of hyperaccumulator plant could increase through boosting the expression of the glutathione gene. Under the regulation of glutathione content, vacuoles still isolate a large amount of ionic cadmium and maintain the overall homeostasis by preferential consuming the resources in the cell sap. These results could provide a further insight of the mechanism of vacuolar compartmentalization and deliver guidance for considering the resource consumption in cell sap and the effect of the glutathione regulation, when we design the genetic hyperaccumulator plants in the future.

## Figures and Tables

**Table 1:** Basic physical and chemical properties and heavy metals content of the soil.

Soil parameter	Value
pH	6.45 ± 0.11
Organic matter content (g kg^−1^)	0.41 ± 0.06
Total N (g kg^−1^)	8.76 ± 0.83
Available P (g kg^−1^)	0.43 ± 0.04
Available K (g kg^−1^)	0.89 ± 0.08
CEC (cmol kg^−1^)	12.03 ± 0.73
Total Cd (mg kg^−1^)	0.3 ± 0.01

**Table 2:** Proportion of glutathione in protoplast and vacuole.

Treatment	Cd0	Cd0 + BSO	Cd20	Cd20 + BSO	Cd40	Cd40 + BSO
Vacuole T-GSH/protoplast T-GSH	34.82%	33.11%	52.38%	52.31%	55.38%	55.29%
Vacuole GSSG/protoplast GSSG	27.72%	27.52%	34.53%	26.91%	26.86%	35.09%
Vacuole GSH/protoplast GSH	39.7%	38.71%	67.43%	79.72%	83.38%	69.44%
GSH/T-GSH (vacuole)	67.62%	58.35%	69.78%	73.22%	76.01%	73.91%

## Data Availability

The data used to support the findings of this study are available from the corresponding author upon request.

## Abbreviations

GSH: Glutathione
ROS: Reactive oxygen species
GSSG: Oxidized glutathione
BSO: Buthionine sulfoximine.
